# Detection of Inflatable Boats and People in Thermal Infrared with Deep Learning Methods

**DOI:** 10.3390/s21165330

**Published:** 2021-08-06

**Authors:** Marcin Łukasz Kowalski, Norbert Pałka, Jarosław Młyńczak, Mateusz Karol, Elżbieta Czerwińska, Marek Życzkowski, Wiesław Ciurapiński, Zbigniew Zawadzki, Sebastian Brawata

**Affiliations:** 1 Institute of Optoelectronics, Military University of Technology, 2 Gen. S. Kaliskiego St., 00-908 Warsaw, Poland; marcin.kowalski@wat.edu.pl (M.Ł.K.); jaroslaw.mlynczak@wat.edu.pl (J.M.); mateusz.karol@wat.edu.pl (M.K.); elzbieta.czerwinska@wat.edu.pl (E.C.); marek.zyczkowski@wat.edu.pl (M.Ż.); wieslaw.ciurapinski@wat.edu.pl (W.C.); zbigniew.zawadzki@wat.edu.pl (Z.Z.); 2 Vortex Sp. z o.o., 28/6. Grunwaldzka Ave., 80-229 Gdańsk, Poland; brawata@vortex.mil.pl

**Keywords:** thermal infrared, deep learning, neural networks, automatic object detection

## Abstract

Smuggling of drugs and cigarettes in small inflatable boats across border rivers is a serious threat to the EU’s financial interests. Early detection of such threats is challenging due to difficult and changing environmental conditions. This study reports on the automatic detection of small inflatable boats and people in a rough wild terrain in the infrared thermal domain. Three acquisition campaigns were carried out during spring, summer, and fall under various weather conditions. Three deep learning algorithms, namely, YOLOv2, YOLOv3, and Faster R-CNN working with six different feature extraction neural networks were trained and evaluated in terms of performance and processing time. The best performance was achieved with Faster R-CNN with ResNet101, however, processing requires a long time and a powerful graphics processing unit.

## 1. Introduction

The eastern border of the European Union often runs through wild winding and meandering rivers located in hard-to-reach wooded or mountainous areas like the Bug River in Poland or the Prut River in Romania. The marshy banks of these rivers are covered with trees and bushes and the water level can change significantly. Despite being a natural border, these rivers are often destinations for smuggling people, drugs, and cigarettes into the EU [1]. Due to the significant price difference, cigarette smuggling is a particularly common case causing large financial losses. Such smuggling usually takes place with the use of several-meter-long inflatable boats, in which 1 or 2 people transport packages. The packages are picked up from the boat by people on the shore and immediately hidden in the bushes for later collection.

The fight against such smuggling activities is usually based on intelligence information combined with the use of electro-optical observation heads equipped with acoustic sensors, infrared, and visible light cameras [2,3,4,5]. The heads are mounted on stationary towers or mobile platforms temporarily observing a selected area. In both cases, monitoring from a distance of several kilometers requires expensive highly-sensitive long-range imagers as the suspected activities are detected mainly by an operator. Currently, the wide market offers relatively inexpensive short- and medium-range cameras, which allows using several cameras successively to observe key sections of the river several hundred meters long. In this case, intruders in a boat can be detected early on when they cross the border, which is predominantly defined by the river midline. However, this approach requires an automatic boat detection software that is likely to distinguish the boat from other floating objects such as tree branches or waves on the water. 

Visible light cameras offer higher detectability due to higher spatial and image resolution, but their performance decreases under low visible light conditions. Infrared illuminators can slightly improve the situation, but usually they have a short range and can be easily detected by smugglers. On the other hand, thermal cameras usually provide images of lower spatial and pixel resolution than visible light cameras, but they can operate in any lighting conditions. Under moderate lighting conditions, contrast of the boat in the river can be higher with thermal cameras since the water surface reflects the low-temperature sky radiance. Considering the above rationales, we selected thermal cameras as the optimal for further studies.

Commercially available thermal cameras are sometimes equipped with embedded intelligent video analytics (so-called edge computing) which can automatically detect various events. They can reliably detect, track, and analyze objects, as well as alert an operator when a predefined alarm is triggered [6,7,8]. The alarm rules include detection of an object in the selected area, crossing the selected line, or entering/leaving the selected area. The object can be filtered considering its size, aspect ratio, speed, movement direction, and duration of an event. Due to the limited capabilities of the processors built into cameras, such analytics are usually based on a smart comparison of subsequent images in the video stream and an analysis of differences between them. They cannot distinguish, among others, floating trees from the boat. Moreover, if the person or boat is stationary, the alarm is not triggered. On the contrary, the methods used in the presented study analyze each image individually and look for specific shapes present during CNN training.

Object detection as a research field has been intensively studied with a variety of approaches being proposed. The introduction of machine learning algorithms resulted in some novel object detection methods. Several various descriptors were proposed which differ in methodology and performance. Appearance-based methods, such as principal component analysis (PCA) [9], linear discriminant analysis (LDA) [10], and independent component analysis (ICA) [11], which project an object into a subspace, have reached high popularity. Other methods including local binary pattern (LBP) [12,13], Gabor jet descriptors [14], histograms of Weber linear descriptor features [15,16], and histograms of oriented gradients (HOG) [17] propose the local-matching approach. These methods create an object representation based on its image divided into blocks. Finally, global matching approaches such as scale invariant feature transform (SIFT) and speeded-up robust features (SURF) [18,19] are used. 

Increasing computational performance of current graphic processing units (GPU) resulted in increasing applicability of neural networks for detection and classification of objects. Most of these architectures rely on convolutional neural networks (CNN). These CNN architectures include: (1) single pass approaches performing detection within single step (single shot multi-box detector (SSD) [20], YOLO [21]) and (2) region-based approaches exploiting a bounding box proposal mechanism prior to detection (faster regional-CNN (Faster-RCNN) [22], region-based fully convolutional networks (R-FCN) [23], PVANET [24], Local R-CNN [25]). Since single pass networks are considered very fast, they sometimes suffer from low detection performance. Region-based networks achieve high accuracy but are reported to be significantly slower than single-shot networks. After proper training, the algorithms can process the video stream frame by frame and classify the detected objects. State-of-the-art CNNs have proven their superiority in a variety of image-based object classification tasks, including autonomous driving on roads [26]. Considering water surface applications, mainly in the field of autonomous navigation and collision avoidance for marine vessels [27], there are some papers dealing with DNNs (deep neural networks) for automatic detection and classification of objects. Scholler et al. [28] comparatively studied three architectures of DNNs (RetinaNet, YOLO, Faster R-CNN) to investigate their performance for ship classification. For the same task, Kim et al. [29] proposed a probabilistic method using Faster R-CNN, Leclerc et al. [30] applied pretrained CNNs based on the inception and ResNet architectures, while Dao et al. [31] used AlexNet deep convolutional neural network. Object detection in thermal infrared imagery finds various applications including detection of human subjects from UAVs [32] and autonomous driving [33,34] and detection capability enhancement [35]. Since many works rely on known detection frameworks [32,34,35,36], new methods directly suited for thermal infrared imaging are introduced [33]. Dai et al. proposed TIRNet, a CNN for object detection in thermal infrared images for autonomous driving. The network uses VGG to extract features and so-called residual branches in the training stage. The TIRNet is fast but the detection accuracy decreases in case of small objects.

Compared with the above applications, we consider a scenario with only two classes of objects (inflatable boats and people) that need to be detected with high probability in various external conditions and distinguished from natural disturbances such as floating tree branches or waves on water. In this paper, we focused on two types of methods—the region proposal network (Faster R-CNN) and the single pass network (YOLO) with different configurations. YOLO has gained several improvements through the years. For this study, YOLOv2 and YOLOv3 have been selected. YOLOv4 [36], which offers improved speed and detection accuracy was not considered in this study since it was introduced a year after the start of this project. To the best of our knowledge, such networks have never been used in such an application. 

The study presents the results of the analysis of three state-of-the-art algorithms for detecting small boats on the river and people in difficult, changing conditions. The selected deep learning methods are meant to combine real-time processing capability with high detection probability and low false detection rates. The study is concluded with an evaluation of the performance results and analysis of the processing methods.

The paper is organized as follows. Description of experiments is provided in Section 2. Section 3 introduces the selected algorithms, namely, YOLOv2, YOLOv3, and Faster R-CNN working with different feature extraction neural networks and describes the development process. Discussion on performance evaluation of the algorithms with regard to selected parameters is included in Section 4. Section 5 presents the summary of the study.

## 2. Experimental Conditions

**Figure 1:**
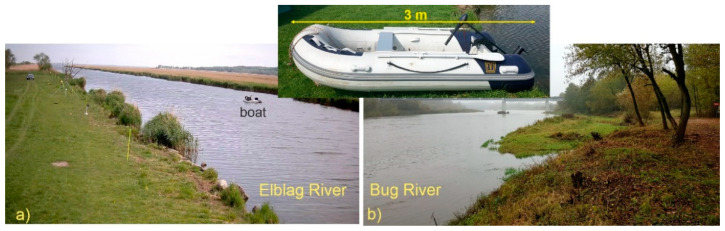
Photos of the test sites on the Elblag (**a**) and Bug (**b**) rivers. Inset shows a photo of the inflatable boat used for tests.

To develop the algorithms, we acquired a large dataset of thermal images presenting a wide range of situations and conditions. All experiments were carried out in two testing fields on the Elblag and Bug rivers in Poland (Figure 1). For tests we used 3-m-long inflatable oars and a motor-powered boat (pontoon) designed for two individuals–Figure 1 inset. The people wore normal clothes adapted to the ambient temperature. 

We considered the following scenario, which is very popular among the smugglers. A boat with packages of contraband flows from one bank to the other at various distances, typically between 50 and 200 m. The packages are collected from the boat by a person waiting on the shore. Next, the packages are hidden in the bushes for later pickup.

The first tests were carried out in June on the 45-m-wide Elblag River in the northern part of Poland. The fairly regular banks of the river (Figure 1a) were partly covered with reeds with a maximum height of 2 m and the river was adjacent to a flat meadow. The water current was of about 1 m/s, while its temperature was equal to 18 °C. Air temperature was about 15 °C at night and about 20–25 °C during the day. The sky was partly cloudy with no rain. The wind was rather weak in the range of 10–20 km/h. Visibility was very good; no fog was noticed.

The second field tests took place on the approximately 100-m-wide Bug River at the eastern border of Poland. Here, the river’s wild and diverse banks were partly covered with reeds, bushes, and trees (Figure 1b) and the river was adjacent to the forest. These tests were performed in April and October. 

During the trials in October, the water current was about 2–3 m/s, while its temperature was equal to about 12 °C. The air temperature was about 8 °C at night and about 12–18 °C during the day. The sky was partly cloudy and sometimes there was a little precipitation. The wind was rather weak in the range of 10–20 km/h. Visibility was quite good, although a slight fog was noticed.

During the two-week trials in April, the weather was rainy with air temperatures between 3 and 8 °C and the water temperature of about 4 °C. The water current was of about 3 m/s. The sky was partly cloudy and the wind was rather weak in the range of 10–20 km/h. High air humidity was noticed.

Tests on the Elblag River lasted 5 days, where 120 crossings at various distances–80 in daylight and 40 at night—were recorded. A typical crossing (one-way) lasted about 30 s. Tests on the Bug River lasted 4 days. Due to the fact that the state border, which could not be crossed, runs in the middle of the river, we only reached the middle of the river and turned back. We recorded 90 crossings at various distances—70 in daylight and 20 at night. Since the water current was faster in the Elblag River, the typical crossing time was longer (about 50 s) and the boat had to maneuver more. 

**Figure 2:**
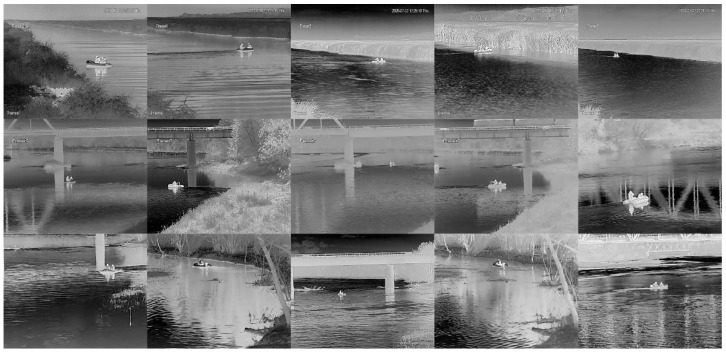
Examples of thermal images of the boat at night (columns 1 and 2) and during the day (columns 3–5) on the Elblag (row 1) and Bug Rivers (row 2—October, row 3—April), respectively. Resolution is of 640 *×* 512.

**Figure 3:**
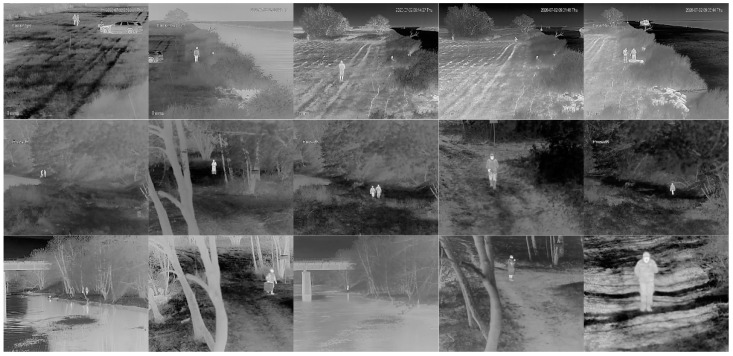
Examples of thermal images of the people at night (columns 1 and 2) and during the day (columns 3–5) on the Elblag (row 1) and Bug rivers (row 2—October, row 3—April), respectively. Resolution is of 640 *×* 512.

In both tests, the camera was placed on the shore on a table situated on a 4-m-high pole, a few meters from the river. For the tests, we used the DH-TPC-BF2221-B7F8 pant tilt dome hybrid camera developed by Dahua [37]. It consisted of visual and thermal imagers enclosed in a robust outdoor housing. The thermal camera features a VGA resolution of 640 × 480 pixels at 30 fps and is based on an uncooled vanadium oxide microbolometer with a pixel pitch of 17 µm and a thermal sensitivity (NETD) of 40 mK. The camera was equipped with a focus-free athermalized 50 mm lens which provided a field of view (FoV) of 12.4° horizontally and 9.9° vertically. According to the datasheet, under ideal conditions, the detection/identification range of a 1.8 × 0.5 m people is of 1471/180 m, while a 4 × 1.4 m vehicle-like object can be detected/identified up to 3268/420 m. Video streams were compressed using the H.264 codec. The thermal camera worked in the auto-scale mode which means that the temperature range in the image was adopted automatically. The thermal imaging camera was integrated with Dahua DH-PFM861-B300 microwave perimeter surveillance radar, which could effectively detect boats and people within a range of up to 200 m. When the radar detected the target, its x-y coordinates were sent to a camera which converted them into pan-tilt coordinates and automatically aimed the camera at that point. Since we worked with a focus-free camera, the observed scene was wide, especially for distant targets, and varied (different azimuth and elevation angles). Figure 2 and Figure 3 present examples of thermal images of a boat with two people crossing the rivers, as well as people walking along the shores at night and during the day, under various conditions and locations, respectively. There are variations in the intensity of people, boats, rivers, and surroundings mainly due to changes of ambient temperature and the position of the Sun. High variability of the scene of the recorded images requires the use of many different images for the correct learning of neural networks.

## 3. Algorithms

Object detection is designed to automatically determine the size and coordinates of objects of interest in an image. We selected three algorithms presenting two different approaches to object detection. The study concerns YOLOv2, YOLOv3, and Faster R-CNN working with different feature extraction neural networks. YOLOv2 and YOLOv3 are real-time processing object detection methods, while Faster R-CNN represents a region-proposal approach and is known as highly efficient but computationally complex [21,22]. 

### 3.1. YOLO

In YOLOv2 and YOLOv3, the object detection process relies on a single convolutional network that simultaneously predicts multiple bounding boxes and class probabilities for those boxes [21]. The YOLO framework considers object detection as a single regression problem, straight from the image pixels to the coordinates of bounding box and class probabilities. The network uses batch-normalization to normalize the output of hidden layers and anchor boxes (v2) or residual blocks (v3) to predefine the bounding box size, thus, improving detection performance. The classification is done with independent logistic classifiers to calculate the likeliness that the input belongs to a specific label. It predicts all bounding boxes in all classes simultaneously for an image. During image processing, the image is taken globally to make predictions. The main improvement in the third version of the algorithm concerns the detection of small-scale objects. The detection is done by applying 1 × 1 detection kernels on feature maps of three different sizes at three different places in the network. 

During the training, YOLO learns the generalizing representations of objects. The second version of YOLO introduced anchor boxes which allowed improving of the algorithm performance while maintaining the processing speed. The anchor boxes must be calculated before the training for the training dataset. YOLOv3 uses residual blocks instead of anchor boxes which allowed for more variants of the scale of objects.

### 3.2. Faster R-CNN

The Faster R-CNN algorithm is based on the idea of a region proposal network (RPN) which generates an object-oriented score for many of the proposed bounding boxes. The initial bounding boxes indicate whether the image section selected contains a background or a foreground object. All the boxes are examined by a classifier and a regressor to check for the presence of objects. The Faster R-CNN is composed of two networks: a region proposal network (RPN) for generating region proposals and a network using these proposals to detect objects. This algorithm, similarly to YOLO, uses anchor boxes, however, they are generated automatically, not predefined before training. It uses a bank of k2 position-sensitive score maps for each category. Those features are calculated by the last convolutional layer. 

### 3.3. Feature Extraction Methods

Faster R-CNN as well as YOLO may use several backbone networks for feature extraction. We have selected a common set of feature extraction networks for YOLO and Faster R-CNN including GoogLeNet [38,39], ResNet18 [40], ResNet50 [40], and ResNet101 [40]. Both GoogLeNet and ResNet networks are state-of-the-art networks providing high performance in classification tasks. 

ResNet is a set of neural networks proposed as a solution for training very deep networks by using so-called identity shortcut connections. Typical ResNet models are implemented with double- or triple-layer skips that contain nonlinearities (ReLU) and batch normalization in between. The network constructs pyramidal cells in the cerebral cortex during data processing. ResNet networks are proposed in many variants with different number of layers and identity connections. During this study, three variants, namely, ResNet18, ResNet50, and ResNet101, were investigated. These three networks bring a wide spectrum of ResNet networks capabilities. 

GoogLeNet is an example of so-called inception networks. GoogLeNet introduced Inception layers which replaced fully connected network architectures. Each inception layer is a combination of a 1 × 1 convolutional layer, a 3 × 3 convolutional layer, and a 5 × 5 convolutional layer with their output filter banks concatenated into a single output vector forming the input of the next stage. The idea of an inception network aims to reduce redundancy or unnecessary activations, thus it improves performance. Moreover, the inception network finally replaces the fully connected layers with a global average pooling which averages the channel values across the 2D feature map, after the last convolutional layer. This operation reduces the total number of parameters and makes the network less prone to overfitting.

### 3.4. Implementation

The selected algorithms require large datasets of thermal images for training purposes. The greater the variety of objects, the better trained and resistant to interference the network will be. Here, neural networks were trained with a set of images presenting objects of interest. Since no publicly accessible datasets are available to properly carry out this phase, we registered a series of video sequences for different weather conditions, distances, and locations. Two classes of objects were selected for the scenario under consideration: a boat with two people floating on a river and a man walking on the shore. 

**Figure 4:**
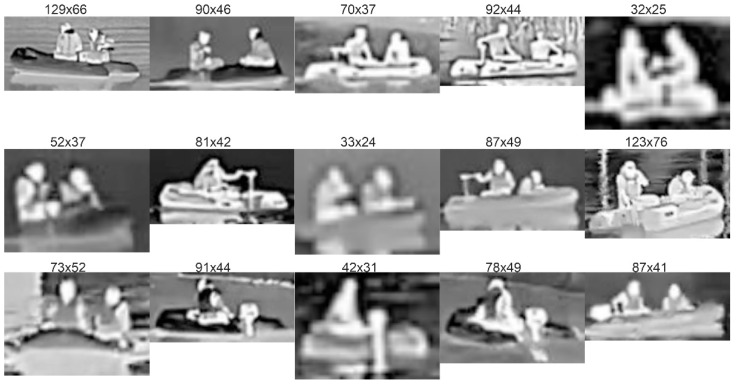
Examples of thermal images of the boat at night (columns 1 and 2) and during the day (columns 3–5) on the Elblag (row 1) and Bug rivers (row 2—October, row 3—April), respectively. The numbers above the image indicate its number of pixels.

**Figure 5:**
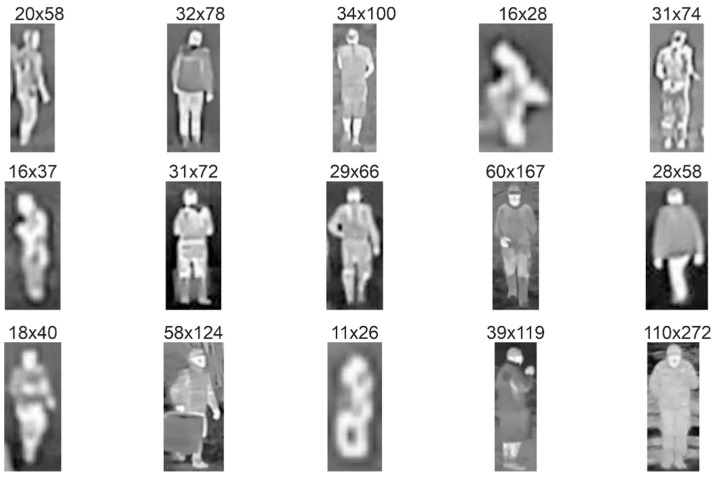
Examples of extracted thermal images of the people at night (columns 1 and 2) and during the day (columns 3–5) on the Elblag (row 1) and Bug rivers (row 2—October, row 3—April), respectively. The numbers above the image indicate its number of pixels.

**Figure 6:**
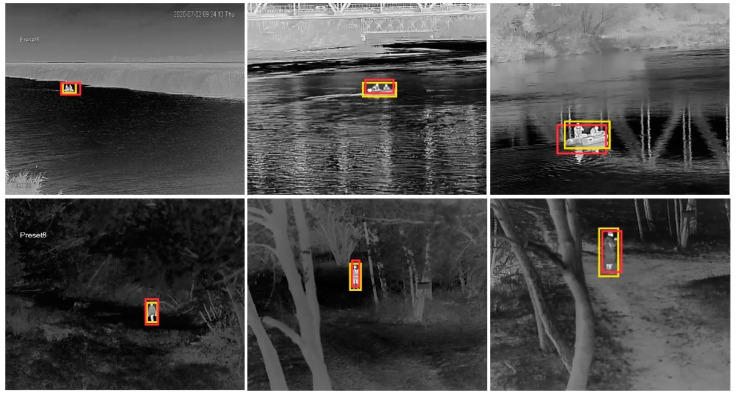
Examples of fitting bounding boxes for IoU metric. The red rectangles indicate the predicted boxes, while the yellow rectangles show the ground truth boxes.

For further training of the networks, we carried out the following procedure. First, from the recorded video streams with a resolution of 640 × 480 pixels we selected 2500 images with targets containing an equal number of objects from both analyzed classes. The selection of images was made to ensure a wide range of scenes and variety of distances between the camera and the objects (see Figure 2 and Figure 3). Afterwards, we manually extracted rectangular images of boats and people from the original images, adding a few pixels on all sides. As a result, images were provided with a resolution in the range from 10 × 25 up to 130 × 70 pixels, often defined as the ground truth bounding boxes (Figure 4, Figure 5 and Figure 6). 

During the experiments, two people were always on the boat for safety reasons imposed by the regulations although in a real situation there is usually only one person on the boat. However, in some views of the boat, one person was obscuring another, thus only one subject was visible in an image as shown in Figure 4 (row 3, columns 2–4). Thus, a diverse set of 2500 boat and people images were created, further used for the DNN training and testing. Figure 4 and Figure 5 present the images extracted from the original images shown in Figure 2 and Figure 3, respectively.

During collection of images presenting subjects on the river’s shore, there were up to 5 walking subjects in various configurations. Subjects were moving in various scenarios, including grouped and distributed persons, at various distances from the camera. 

Finally, this dataset was divided into training and test subsets, respectively. The training–test split ratio was set at 75% and 25%, respectively. The training dataset consists of 1875 images presenting equal number of both objects of interest. All the models used during this study have been pre-trained on ImageNet database. To achieve a higher variability of data, augmentation (mirroring) was applied. For the training process, we set up a threshold for training accuracy up to 0.95. Each algorithm was trained with a number of epochs between 50 and 100, depending on how fast the threshold was achieved during the training phase. All algorithms were implemented and tested in the MATLAB 2021a environment with a workstation equipped with an NVIDIA RTX 2080 graphics processing unit and 64 GB of RAM. 

## 4. Results and Discussion

The selected algorithms, namely, YOLOv2, YOLOv3, and Faster R-CNN, working with different feature extraction neural networks, were tested using the testing dataset composed of 625 images representing different thermal images of boats and people under different experimental and environmental conditions. All algorithms were evaluated with regard to the following parameters: detection rate and false detection rate, classification rate, intersection over union metric, and processing time using GPU and CPU. Table 1 presents the average values determined for various conditions and distances in the range of 50–200 m.

Figure 6 shows the examples of images from the testing set showing both classes of objects at short, medium, and long distances in the range of 50–200 m. The predicted bounding box (red) and the ground-truth bounding box (yellow) are marked for comparison and determination of the IoU metric (see Section 4.3). 

### 4.1. Detection Rate and False Detection Rate 

Detection rate (DR) determines the percentage of the objects correctly detected in the testing dataset, while false detection rate (FDR) describes the percentage of false alarms raised while evaluating an algorithm at the testing dataset. The threshold for IoU used to consider a detection matching the ground truth was set to 70%.

The results presented in Table 1 indicate that Faster R-CNN outperforms other methods. These results clearly show that it may deliver high performance, but DR and FDR vary depending on a feature extraction method and the highest performance was obtained for Resnet101 (DR = 83% and FDR = 4%). 

Performance of YOLOv3 and YOLOv2 is almost similar with DR in the range of 50–65%. Detection rates show the same trend for both YOLOv2 and Faster R-CNN, with ResNet101 as the best performing feature extraction network. Surprisingly, all ResNet networks in combination with the YOLO framework perform better when having less layers. However, both algorithms suffer from a relatively high, unacceptable for practical applications, level of the false detection rate (10–18%). 

### 4.2. Classification Rate

Classification rate (CR) is the ratio of the number of correctly classified objects to the total number of correctly detected objects. Here, all algorithms show high CR value in the range of 81–95%. Faster R-CNN with Resnet101 slightly outperforms other methods, while the second place, with CR = 91%, belongs to YOLOv2 with GoogLeNet, as well as to Faster R-CNN with ResNet50. 

### 4.3. Intersection over Union

During the study, we used the intersection over union (IoU) metric, which is mostly used to measure the accuracy of object detectors [41]. IoU describes how well the predicted bounding box fits to the ground truth bounding box. This metric determines the percentage of overlap between a predicted bounding box (red) and a ground-truth bounding box (yellow) as presented in Figure 6. The ratio of the common part of the areas of two rectangles (yellow and red boxes) to the sum of these two areas defines IoU, as well as the detection accuracy. 

One can notice that Faster R-CNN with ResNet101 reaches IoU = 79% and outperforms other methods. The performance of YOLOv3 is approximately 20–30 percentage points higher than YOLOv2 and reaches similar values to Faster R-CNN with other feature extraction networks. 

IoU metric is important for the theoretical comparison of the methods, but from the operation point of view, even finding a small part of the object is satisfactory. Correct detection, indication of the place, and classification are more important than a high value of IoU.

### 4.4. Processing Time

Table 1 presents the average image frame processing time for each of the algorithms investigated during this study. The values were achieved for two processing units–CPU (central processing unit) and GPU (graphics processing unit). We used an NVIDIA RTX 2080 and Intel Core i7-8700K as GPU and CPU, respectively.

Faster R-CNN, the best method in terms of performance parameters, requires the powerful GPU to process the single image frame in the time range of 0.5–0.7 s regardless of configuration. This algorithm may also operate with a high-power CPU; however, the processing time is unacceptably high (15–25 s) for real time processing systems. 

On the other hand, the single image frame processing time achieved by YOLOv2 and YOLOv3 is relatively short. When performing the GPU-based processing, even 15–30 frames per second (fps) can be achieved. Moreover, these algorithms also allow for fairly fast processing of images using the CPU, reaching the processing rate of about 1–2 fps.

### 4.5. Discussion on Real Life Applications

Based on Table 1, it can be concluded that the price for outperforming results of Faster R-CNN-based algorithms is a longer processing time. On the one hand, the performance of both YOLO-based algorithms in terms of DR, FDR, CR, and IoU is lower by about 20, 6, 5, and 10 percentage points, respectively, in comparison to the best Faster R-CNN with ResNet101. On the other hand, the processing time of YOLOv3 is about 10 and 30 times shorter for GPU and CPU, respectively. 

Hence, two opposite approaches, both based on GPU, can be considered. Implementation of Faster R-CNN with ResNet101 provides high detection and classification rates, but with a low processing time of 1 fps. YOLOv3 with DarkNet53 should achieve a medium detection and classification performance but with a high, up to 25 fps, real-time processing speed. 

In practice, the selection of the right algorithm depends on many parameters, including distance to the objects, exposure time (i.e., time, when the boat or people are visible in the camera FoV), and variability of external conditions. For shorter distances, up to about 100 m, detection and classification rates will be higher than the mean values presented in Table 1. Therefore, YOLOv3 may offer a higher, possibly acceptable, performance. On the other hand, for higher distances, probably only Faster R-CNN can provide the acceptable yield. 

Let us assume that the typical exposure time (e.g., time of crossing the river) is 30 s. During this time, YOLOv3 is able to process 750 images, while Faster R-CNN only 30. In the scenario under consideration, it is not necessary for all image frames to give the correct results. Even if one in ten images is correct, it should be enough to detect subjects or boats. In the next phase of our project, we plan to evaluate the developed algorithm over a longer period of time, for various scenarios and environmental conditions. Based on our previous results and experience, it is expected that the detection rate of at least one object (boat, person) per event will be around 95/90% for boats up to 100/200 m and 90/85% for a single person up to 100/200 m, respectively. 

Analysis of Figure 4 and Figure 5 clearly shows that the visibility of objects, even for a similar pixel resolution depends on environmental conditions, including temperature, insolation, and transmission of the atmosphere. Thus, in areas with higher variability of environmental conditions, more reliable algorithms with higher detection rates should be applied. 

## 5. Summary

This study reports on the detection of small inflatable boats and people in various difficult terrains of wild rivers from a distance of 50–200 m using deep learning algorithms in the thermal infrared domain. Three acquisition sessions were carried out during spring, summer, and fall under various weather conditions that resulted in a selection of 2500 images. The images were next used to train and test three state-of-the-art deep learning algorithms, namely, YOLOv2, YOLOv3, and Faster R-CNN working with six different feature extraction neural networks. 

The algorithms were evaluated in terms of performance parameters and processing time. The best performance was achieved with Faster R-CNN with ResNet101, which, however, suffers from high processing time (0.5–0.7 s) even for the powerful GPU. The performance of both YOLO-based algorithms was lower by about 5–20 percentage points, but their processing times were noticeably faster (about 10 times), which provides real time processing capability. 

In conclusion, the detection of river crossings in small inflatable boats is difficult due to the diverse and changing environmental conditions. Moreover, the detection capabilities are limited by the spatial resolution of the observed scene and the thermal contrast between the objects and the background. The presented analysis shows that the greatest challenge remains the correct detection of the object of interest while maintaining a low false alarm rate. It seems that currently with the use of fairly advanced computers with powerful GPUs, the much-desired high detection rate can only be achieved with the use of complex, resource-consuming, and, therefore relatively slow algorithms. On the other hand, 1-s processing time seems to be sufficient to detect objects within seconds when the protected area is crossed.

## Figures and Tables

**Table 1:** Algorithms’ evaluation results.

CNNs for Feature Extraction	Detection Rate (%)	False Detection Rate (%)	Classification Rate (%)	Mean IoU (%)	Processing Time (s)	
					GPU	CPU
YOLOv2						
GoogLeNet	56	12	91	47	0.04	0.44
ResNet18	51	18	81	41	0.03	0.43
ResNet50	59	10	83	43	0.04	0.49
ResNet101	61	10	90	28	0.06	0.76
YOLOv3						
SqueezNet	61	10	88	66	0.06	0.60
DarkNet53	65	10	90	69	0.07	0.69
Faster R-CNN						
GoogLeNet	71	7	90	60	0.51	16.51
ResNet18	69	7	89	73	0.43	15.43
ResNet50	75	6	91	61	0.53	17.53
ResNet101	83	4	95	79	0.71	24.71

## Data Availability

Not applicable.
